# User Guided Selection and Alignment of Sequence Data by GeneMatrix

**DOI:** 10.1007/s00239-026-10319-2

**Published:** 2026-05-25

**Authors:** Simon J. Goodman, Ian M. Carr

**Affiliations:** 1 School of Biology, Faculty of Biological Science, University of Leeds, Leeds, LS2 9JT UK; 2 School of Medicine, Leeds Institute of Medical Research at St James’s, University of Leeds, Leeds, LS9 7TF UK

**Keywords:** Phylogenetics, Multiple sequence alignments, Software

## Abstract

The alignment of multiple protein or DNA sequences is a common task in many biological fields. These alignments may then be used to identify conserved regions or positions, which could in turn be used to score the likely pathogenicity of a sequence change in a patient, detect functional domains that can determine the activity of an unknown gene, or map evolutionary relationships by tracking changes in conserved regions. The development of high-throughput sequencing technologies has trivialised the generation of sequencing data, which is made available through public repositories, such as GenBank, hosted by the NCBI. In October 2024, GenBank held approximately 4.7 billion sequences from over 580,000 species. Consequently, the constraints of multiple sequence alignment estimation have moved from data generation to data retrieval, extraction and filtering. While human-curated datasets for widely used sequences have been released, retrieving datasets for less commonly used sequences can be onerous. Therefore, we have developed GeneMatrix, an application that aids the extraction, filtering, aggregation, and alignment of DNA and protein sequences from GenBank-formatted files of single gene sequences or genomes such as viruses and mitochondria.

**Supplementary Information:**

The online version contains supplementary material available at 10.1007/s00239-026-10319-2.

## Introduction

Phylogenomic, phylogeographic, population genetic, molecular evolution, and in silico functional genomic analyses rely on compiling curated orthologous sequence datasets from online databases or from de novo sequence assemblies. Tools are needed to streamline the curation and preparation of sequence datasets for downstream analyses, for users without extensive bioinformatics experience, for teaching and other contexts where graphical user interfaces are more convenient and improve accessibility.

To aid the analysis of commonly used sequences, a range of human-curated sequence datasets, such as MIMT (Cabezas et al. 2024), PR2 (Guillou et al., 2012), SILVA (Quast et al. 2013) and ya16sdb (Rosenthal and Hoffman 2019), have been released. However, this type of resource may quickly become obsolete or is focused on specific sequences such as 16S and 18S sequences that, while commonly used to annotate eDNA and microbiome samples, are of limited utility in other biological fields. As a result, in many cases, sequence data must be retrieved from large, heterogeneous archives such as GenBank, whose breadth and long history necessarily result in variable annotation quality and legacy naming conventions.

Workflows typically start with compiling reference sequence sets for target features from online sources such as GenBank. GenBank is a major repository for DNA and protein sequences, holding approximately 34 trillion nucleotides for 4.7 billion sequences from over 580,000 species (Sayers et al. 2022, 2025), with this value expected to double every 18 months (Sayers et al. 2022). GenBank makes this data available in a range of formats, with the preferred format being the GenBank sequence file format. This format is human-readable, containing both the sequence and varying amounts of descriptive meta-data. Since these descriptions are provided by the depositor, there is also considerable variation in their quality (for example misattribution of gene or species labels), level of detail and the nomenclature used, which can complicate curation of target sequences.

Additionally, while highly informative, the structure does not easily allow the manual extraction of sequences related to specific features, such as open reading frames in a genomic sequence or individual genes in a virus or mitochondrial genome sequence. As a result, a number of software packages have been developed, such as the GenBank Feature Extractor webpage (Stothard 2000), Biopython’s SeqIO module (Cock et al. 2009), or the Streaming Sequence Extractor (https://github.com/biobricks/streaming-sequence-extractor) to parse and extract data from GenBank files. Since GenBank Feature Extractor is implemented as a webpage that requires the copying and pasting of input and exported data, it is of limited use for the analysis of moderately large datasets, whereas the others are more suitable for creating larger datasets but must be implemented within a user-developed script. A range of R packages for sequence retrieval, parsing and curation are also available (e.g. *phylotaR* (Bennett et al. 2018), *AnnotationBustR* (Borstein and O’Meara 2018), *genbankr* (Becker and Lawrence 2022)), but require good competency in the R programming language to use effectively. Similar capabilities are available within some standalone software packages such as the MEGA series (Kumar et al. 2024) and PhyloSuite (Zhang et al. 2020) with varying levels of capability. Similarly, the command-line Python script EASER (Maldonado et al., 2013) was designed to download sequences from the Ensembl database for a given gene across a chosen set of organisms.

Once the relevant data has been extracted and saved in sequence-specific FASTA files, it may then be used to generate multiple sequence alignments with programs such as ClustalW (Larkin et al. 2007), PRANK (Löytynoja 2014), MAFFT (Katoh et al., 2013), and MUSCLE (Edgar 2004). Alignments then form the starting point for downstream applications including phylogenetic, phylogeographic, population genetic and molecular evolution analyses, investigation of functional properties of sequences, and primer design.

Workflows for these analyses may require additional steps such as removal of areas of poor quality or ambiguous alignment (e.g. using GBlocks (Talavera and Castresana, 2007), determining the optimal parameters, evolutionary models and analysis schemas for each alignment in phylogenetic analyses (e.g. using PartitionFinder2 (Lanfear et al. 2017)), and concatenating alignments from multiple loci. In this context, the interactive command-line Perl package LMAP_S (Maldonado 2019) automates the detection of outlier sequences and the production and refinement of alignments, ultimately generating phylogenetic trees.

The proliferation of sequence data in repositories like GenBank and the development of numerous multiple-sequence alignment programs such as MAFFT, PRANK and MUSCLE have benefited a wide range of biological disciplines. However, the extraction, filtering and alignment of sequence data is a neglected task that is a non-trivial task for a bench scientist with limited bioinformatics skills. Consequently, we have developed GeneMatrix, an application that, when given a series of GenBank accession IDs, GenBank files, or FASTA files, can extract and collate DNA and/or protein sequences into a series of FASTA files and facilitate their curation by helping the user to identify sequences with misattributed feature names or species designations. GeneMatrix can then automate their alignment using one of four popular aligners, with the optional cleaning of the alignments by GBlocks. Finally, GeneMatrix is able to guide the user through the use of PartitionFinder2 to determine the optimum parameter for any subsequent phylogenetic studies. These functions should help improve the efficiency of preparing datasets, particularly for phylogenomic analyses of organelle genomes and viruses.

## Methods and Materials

The third-party applications MUSCLE, ClustalW, MAFFT, PRANK, GBlocks, PartionFinder2 and Anaconda were obtained as outlined in supplementary Table 1.

### Design and Implementation

**Fig. 1:**
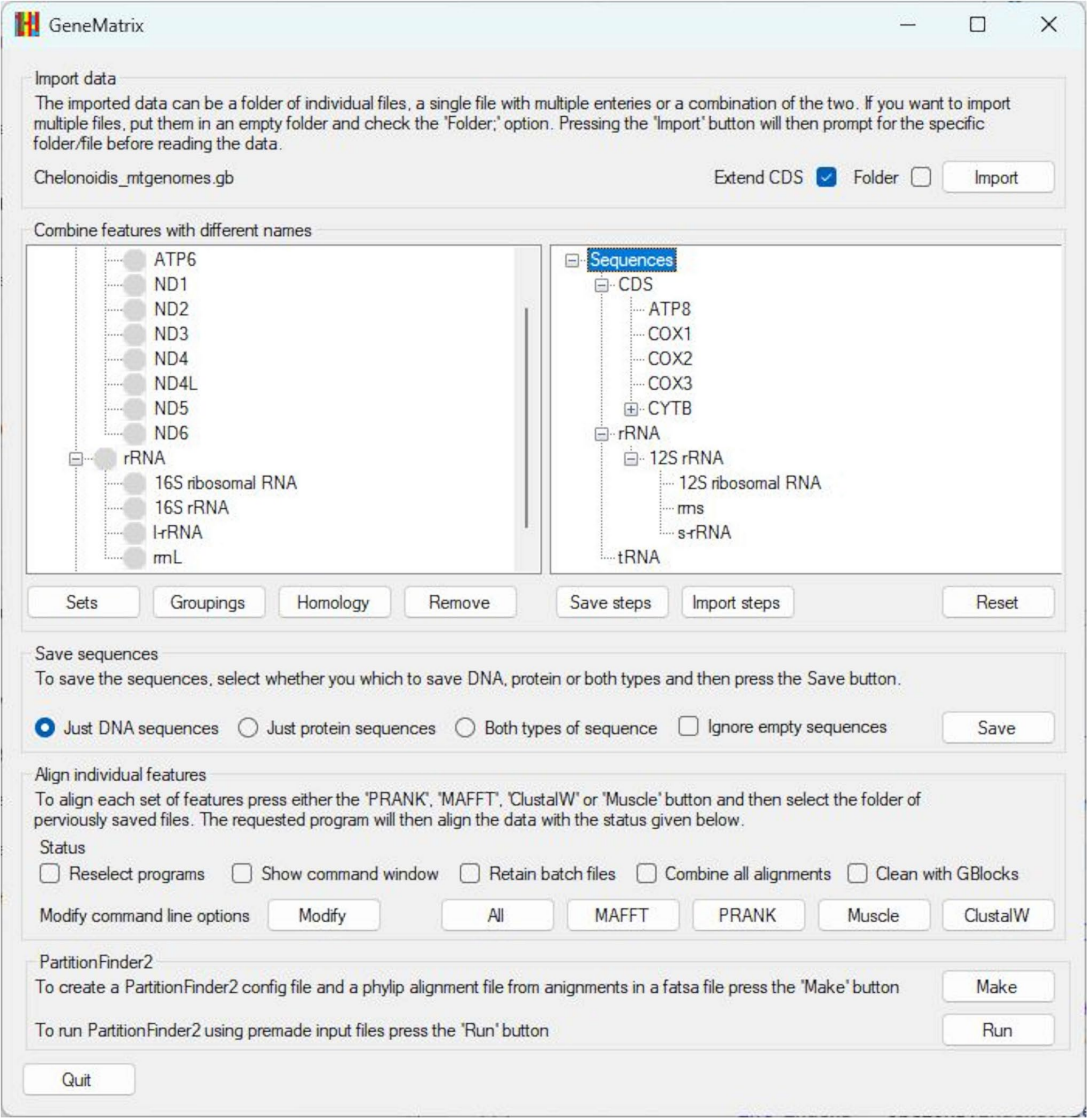
GeneMatrix’s primary window. Genes are shown as nodes in two tree-like structures. Unselected sequences are displayed on the left, while selected sequences are displayed in the right-hand tree. Where a gene is linked to two or more names, the nodes of the alternative names can be added to the node with the preferred name; for instance, the sequences named 12S ribosomal RNA, rrns and s-rRNA are present in the 12A rRNA node. When exported, all sequences in these three nodes will be exported to a single file

**Fig. 2:**
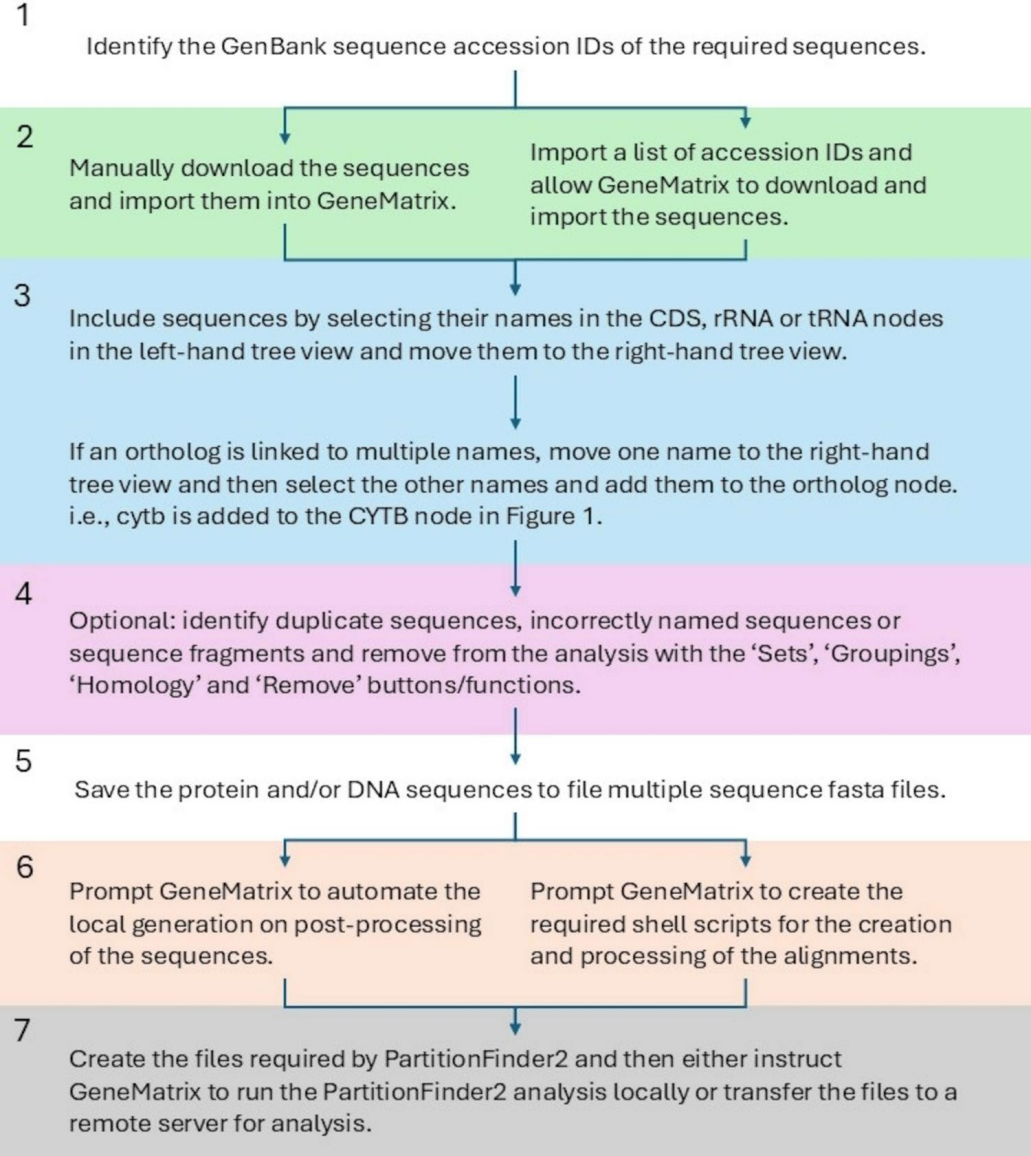
GeneMatrix workflow overview. The workflow proceeds through seven steps: in Step 1, users compile a list of GenBank accession IDs for the required sequences; in Step 2, these sequences are imported either as accession IDs or GenBank files; in Step 3, the required sequences are selected and orthologues aggregated where appropriate; in Step 4, spurious sequences are identified and removed; in Step 5, the curated sequences are saved as multi-sequence FASTA files; in Step 6, multiple sequence alignments are generated; and in Step 7, PartitionFinder2 is directed to determine the optimum parameters downstream phylogenetic analysis

GeneMatrix is developed in C# using the.NET framework. The program consists of a main window with two panels that display the unselected (left-hand panel) and selected (right-hand panel) sequences in a tree-like structure (Fig. 1). The trees include up to four branches for sequences of type CDS, tRNA, rRNA and Unknown. Feature-specific sequences can be selected by clicking on them in the left-hand panel and then clicking on the appropriate branch in the right-hand tree, with only the sequences in the right-hand tree exported and used in any subsequent analysis. A schematic of GeneMatrix’s workflow is shown in Fig. 2. Importing data.

Data can be imported by one of three processes using: a text file listing a number of GenBank accession IDs, pre-downloaded GenBank sequence files, or as a series of FASTA files. Extensively annotated GenBank sequence files are preferred due to the extensive secondary information they contain, making them more flexible than FASTA files.

Importing a list of accession IDs: GeneMatrix will accept a single text file containing a list of GenBank accession IDs, with one entry per line. Once imported, the sequence data for each GenBank accession ID is downloaded from the NCBI site using the URL: https://eutils.ncbi.nlm.nih.gov/entrez/eutils/efetch.fcgi?db=nuccore&id=<accession>rettype=gb&retmode=text

where < accession > is replaced by the relevant accession ID. The data is downloaded as GenBank-formatted text, which is processed on the fly in the same manner as a local GenBank file as described below. However, the NCBI website limits each computer to 3 requests per second, if this is exceeded, subsequent requests are rejected until a suitable period has elapsed. To prevent this, if downloading and processing a GenBank record takes less than 350 ms, GeneMatrix will momentarily pause before requesting the next sequence.

Importing GenBank files: A GenBank file may contain one or more entries and can be imported as either a single file or a folder of files. Each entry is processed in turn by first reading the record’s description and extracting the entry’s ID and source organism. Next, each CDS, tRNA and rRNA feature is processed, noting the name, coordinates and orientation of the linked sequences. It is not uncommon for the start or end of a sequence to be uncertain with any ambiguity marked by a < or > character. In these cases, the sequence’s start and end points are set as the given coordinates, and no attempt is made to determine the actual start and end points. If a CDS feature is linked to a protein sequence, its protein sequence is also retained.

A feature may have one or more tags related to its name, with GeneMatrix searching for the /gene, /product, /protein_id, and /locus_tag name tags. If multiple tags are present, the name is taken from the first annotated tag in the following order of /gene, /product, /protein_id, and finally /locus_tag.

Once the metadata has been processed, the entry’s sequence data is imported, but only the sequence related to a feature is retained. Not all GenBank entries contain data related to the sequence’s features and may be just a sequence; in these cases, no data is retained, and the sequence’s accession ID is displayed in a pop-up window, noting no data was imported for the listed accession ID.

While the official GenBank format (https://www.ncbi.nlm.nih.gov/genbank/samplerecord/) states that a feature’s coordinates reference the feature’s first and last nucleotide, this is often not the case, with the coordinates commonly referring to the position of a codon’s first base. If selected, GeneMatrix ‘s ‘Extend CDS’ option identifies CDS features that lack a start and/or stop codon and determines if they are present in the flanking three bases. If found, the CDS sequence is extended to correct the possible oversight.

Importing FASTA files: GeneMatrix can also import a folder of user supplied FASTA files, for example user-generated mitogenomes or virus genomes, with each file expected to contain a single sequence, with DNA sequences in the forward orientation. It does not differentiate between nucleic acid and amino acid sequences; therefore, FASTA files in a folder must be either all protein or all nucleic acid sequences.

If the sequence’s name contains three semicolons, such as those generated by Mitos, a pipeline for annotation of de novo mitogenome assemblies (Bernt et al. 2013), the name is split into four fragments, with the first used to denote the species of origin and the sequence’s name taken from the fourth part. If the third section is a ‘- ‘ character, the sequence is reverse complemented. When a sequence’s name doesn’t conform to this format, the file’s name is used as the species name, and the sequence’s name will be used as the gene name.

### Selecting the Optimal Data Set

Incorrect annotation of GenBank records is a common issue, with a sequence attributed to the wrong species being a recurrent issue (Bensch et al. 2021). Consequently, it is advisable that the sequences used in the final alignments should undergo several rounds of refinement. Ideally, the initial dataset should include duplicated sequences for each species, which can then be compared to identify incorrectly annotated sequences. To facilitate this, GeneMatrix is able to scan selected DNA sequences linked to a gene and detect identical entries, placing them into gene sets. Where GenBank files contain multiple gene sequences, these can be grouped into supersets where each group contains GenBank files with the same set of gene sequences. Ideally, these sets should demonstrate a one-to-one relationship with a species. Instances where a species is linked to more than one sequence may reflect natural variation within a population or an erroneously annotated sequence. Similarly, when the same sequence is linked to multiple species, this may be due to miss-attribution of a sequence or a high level of conservation between two closely related species.

As well as assigning a sequence to the wrong species, attributing a sequence to the wrong gene family is also a common event (Prada and Boore 2019). Consequently, as well as identifying duplicated sequences, GeneMatrix can perform a Needleman-Wunsch pairwise comparison (Needleman and Wunsch 1970) between all selected sequences, with the score for the best global alignment returned for each pair of sequences. Ideally, this can differentiate between sequences from closely related species and sequences from the same species that differ at several polymorphic positions. The Needleman-Wunsch pairwise alignment scores can also be used to identify outliers by first identifying a common sequence – the sequence with the greatest homology to all the other sequences. Then by comparing other sequences’ global alignment scores to the common sequence and determining the typical degree of deviation in the data set, outliers can be seen as those whose alignment scores are noticeably lower than expected range. These outliers may be either sequence fragments or incorrectly annotated sequences, and once identified, they can be removed by submitting a text file containing the unwanted accession IDs to GeneMatrix.

### Saving Sequences as Gene-Specific FASTA Files

Once data is imported, GeneMatrix displays unique gene names as an array of nodes in the left-hand tree structure. Sequences from a GenBank file are placed in the appropriate CDS, tRNA or rRNA branch, while sequences from the FASTA files are placed in the Unknown branch. To select a series of genes for analysis, click on each node in the left-hand panel and then click on the appropriate destination node in the right-hand tree. Imported sequences for a gene may have one or more names; consequently, a gene may be represented by multiple nodes, each linked to one or more sequences. To aggregate these sequences into a single node, move the node with the preferred name to the right-hand tree, then select the nodes with the alternative gene names, and finally, select the node with the preferred name in the right-hand tree. This will link the sequences with the alternative names to the node with the preferred name, rather than one of the CDS, tRNA or rRNA main branches (Fig. 1). Once the required genes have been selected, the protein, DNA, or protein and DNA sequences can be exported to a series of appropriately named FASTA files in a selected folder.

### Automating the Analysis

GeneMatrix can automate the alignment of the sequences in each of the FASTA files in a folder using MAFFT, PRANK, MUSCLE or ClustalW, followed by the cleaning of the alignments with GBlocks, provided the relevant program is present on the computer. To do this, GeneMatrix prompts the Windows command shell to process a batch script created by GeneMatrix. If required, the resultant alignments can be concatenated to a single multi-sequence alignment file. By default, the alignments are performed using the default/generic parameters suggested by each aligner’s user guide (supplementary Table 2), but these commands can be modified, with any changes retained and applied in subsequent analysis. Alternatively, users can perform alignment of sequence sets externally using an application of their choice and then import alignment files into GeneMatrix for subsequent workflow steps.

While aligned sequences can be used in a variety of workflows, their use in phylogenetic and phylogenomic analyses is a key application. Therefore, GeneMatrix can guide their analysis by PartitionFinder2 to determine the optimum parameters for their subsequent analysis by programs such as Beast (Bouckaert et al. 2019) or MrBayes (Ronquist et al. 2012). Unlike the aligners, which can be run via a single command-line instruction, PartitionFinder2 requires a configuration file containing many user-defined analysis parameters. As a result, automating PartitionFinder2 necessitates a two-step process of first creating the configuration file and then performing the actual analysis. GeneMatrix assists in these steps through two process-specific windows, the first collating the alignment data and parameters needed to create the configuration file, with the second form guiding the selection of the command-line arguments and automating the execution of PartitionFinder2. Since PartitionFinder2 requires the installation of the now obsolete Python 2.7, it is often installed in a Conda environment – GeneMatrix will search for and activate the required Conda environment when automating PartitionFinder2.

## Discussion

The generation of multiple sequence alignments is a crucial step in a wide range of biological disciplines; nevertheless, despite considerable effort dedicated to their production and analysis, the extraction and curation of sequences from databases like GenBank has been overlooked. The exponential expansion of sequences stored in public repositories has replaced the need for sequence generation with the bioinformatics challenges of data selection, extraction and validation. Consequently, we have developed GeneMatrix, an application primarily targeted to the processing of GenBank data files, particularly those for mitochondrial and small virus genomes, or user-generated fasta files for de novo assemblies of such, which contain multiple gene sequences.

While representing a significant resource, GenBank entries suffer from a number of issues arising from a light touch validation system designed for ease of use rather than rigorous compliance to a set standard. This has led to sequences being attributed to the wrong species (Bensch et al. 2021) or the wrong gene (Prada et al., 2019). Consequently, to detect incorrect attribution, GeneMatrix can place selected sequences into sets that should segregate by species. When a set is linked to multiple species, the user should determine if this is due to the conservation of sequence between closely related species or a sequence incorrectly assigned to the wrong species. Similarly, when a sequence is linked to multiple sets, it should be determined if a sequence is incorrectly annotated or if the different sequences represent polymorphic variation within a population. GeneMatrix can also perform a series of Needleman-Wunsch pairwise alignments whose results can be used to identify sequences from the same species that differ at a few polymorphic positions as opposed to sequence fragments or sequences assigned to the wrong gene family.

The absence of an enforced gene naming convention may result in orthologs being named after either a gene name or a gene symbol; these, which in turn, may be misspelt. This is compounded by the use of alternative names and symbols; for example, there have been repeated attempts to standardise the naming of the histone genes (Talbert et al. 2012; Seal et al. 2022). When naming conventions have changed, historical GenBank entries are not necessarily updated to reflect current conventions. This variation in sequences naming makes the automated aggregation of orthologous sequences based on their reported name very difficult. Consequently, GeneMatrix provides a simple user-driven mechanism for the selection and aggregation of gene-specific sequences based on each sequence’s name. While GeneMatrix is primarily designed to create sets of orthologous gene sequences for phylogenetic studies, it can also be used to aggregate paralogous sequences, such as the histone genes.

GenBank entries may also contain truncated sequences due to either the record containing a partial sequence, or the inaccurate reporting of a feature’s coordinates. Little can be done to correct a partial sequence other than to omit them from the exported data set. However, for CDS sequences lacking a start and/or stop codon, it is possible to prompt GeneMatrix to scan the immediate 1—3 bases of flanking sequences to find sequences whose inclusion will add the missing codon. These sequence retrieval, import, validation and curation tools provide additional and complementary functionality, or an enhanced user interface for these aspects relative to other standalone software such as *MEGA* and *PhyloSuite*.

GeneMatrix enables users to generate multiple alignments of selected sequences using one of four different sequence aligners (ClustalW, PRANK, MAFFT and MUSCLE), and to clean those alignments with GBlocks, before determining the optimum parameters for subsequent phylogenetic studies with partitionFinder2. These programs were chosen because they are widely used in the phylogenetics community, are stable, and are supported by extensive documentation. Due to the modular design of GeneMatrix, the subsequent inclusion of other command line aligners would be a relatively trivial task.

The operation of GeneMatrix is split into 3 tasks, the production of the gene/feature specific FASTA files, their alignment and phylogenetic analysis parameter optimisation by PartitionFinder2. Each of these tasks is performed independently of the others and so allowing GeneMatrix to be used in conjunction with other software. For example, GeneMatrix could be used to generate the sequence specific FASTA files, that are then aligned using user specific scripts and the determination of optimal parameters for subsequent analysis performed with LMAP_S. proliferation of sequence.

For very large datasets, it may be sensible to create the multiple sequence alignments on a high-performance server rather than a desktop computer. However, since current computers have significant processing power, they can be used to align over 70 sequences of over a kilobase in length in less than 10 min. This makes GeneMatrix’s ability to automate the creation of a series of multiple sequence alignments very useful. While the specific time required to create a set of alignments depends on the sequences and the computer, most computers could easily process the DNA and protein sequences for the 13 CDS and 2 rRNA genes present in the 70 mitochondrial genomes present in the Chelonoidis_mtgenomes.gb file located on GeneMatrix’s GitHub repository page overnight.

In summary, while sequences can be manually extracted, filtered and queued for alignment, the process can be time-consuming and error prone. Therefore, the ability of GeneMatrix to streamline these processes and then automate the alignment process, the cleaning of the alignments by GBlocks, and parameter selection using PartitionFinder2 is a significant aid to researchers in a range of biological disciplines.

## Supplementary Information

Below is the link to the electronic supplementary material.

Supplementary file1 (DOCX 19 KB)

## Data Availability

GeneMatrix source code, executable files and used guide are available from its GitHub repository: https://github.com/msjimc/GeneMatrix.
