# Supplementary material for: User Guided Selection and Alignment of Sequence Data by GeneMatrix
Source: J Mol Evol. 2026 May 25;94(3):541–8. doi: 10.1007/s00239-026-10319-2 (PMC13294245; doi:10.1007/s00239-026-10319-2)
Supplement: Supplementary file 1 — Supplementary file1 (DOCX 19 KB) [file 239_2026_10319_MOESM1_ESM.docx]

Supplementary Tables

| Application | Version used | Binary or installation File | Website and manual |
| --- | --- | --- | --- |
| Anaconda | 2024-10-1 | Anaconda3-2024.10-1-Windows-x86_64.exe | Website: https://www.anaconda.com/  Manual: https://docs.anaconda.com/anacondaorg/user-guide/ |
| Clustal | 2.1 | Clustalw2.exe | Linux Binaries: https://github.com/coldfunction/CUDA-clustalW  Windows binaries https://github.com/msjimc/GeneMatrix |
| GBlocks | 0.91 | Gblocks_Windows_0.91b.zip | Website: https://molevol-ibe.csic.es/Gblocks.html Manual: https://www.biologiaevolutiva.org/jcastresana/Gblocks/Gblocks_documentation.html |
| MAFFT | 7.526 | Mafft-7.526-win64-signed.zip | Website: https://mafft.cbrc.jp/alignment/server/index.html  Manual: https://mafft.cbrc.jp/alignment/software/manual/manual.html |
| Muscle | 5.3 | Muscle-win64.v5.3.exe | Website: https://www.drive5.com/muscle/  Manual: https://drive5.com/muscle5/manual/cmd_align.html |
| PartitionFinder2 | 2.1.1 | Partitionfinder-2.1.1.zip | Website: https://www.robertlanfear.com/partitionfinder/  Manual: https://www.robertlanfear.com/partitionfinder/assets/Manual_v2.1.x.pdf  Tutorial: https://www.robertlanfear.com/partitionfinder/tutorial/  GitHud: https://github.com/brettc/partitionfinder/releases/tag/v2.1.1) |
| PRANK | 140603 | Prank.windows.140603.zip | Website: https://ariloytynoja.github.io/prank-msa/  Manual: https://ariloytynoja.github.io/prank-msa/#main-program-options |

Table 1: Location websites and manuals for third-part applications automated by GeneMatrix

| Application | Command and description |
| --- | --- |
| ClustalW | clustalw2.exe -INFILE=[input file] -TYPE=[DNA or PROTEIN] -OUTPUT=FASTA -OUTFILE=[results file]  where:   - [input file] is the fasta file to align. - [DNA or protein] is the type of sequence to align. - [results file] is the name of the file to save the alignment to. |
| MAFFT | mafft --auto --retree 2 --inputorder [input file (Linux)] > [results file (Linux)]  where:   - --auto: prompts MAFFT to use the best options for the alignment: from L-INS-i, FFT-NS-i and FFT-NS-2, according to data size (see its website for details). - --retree 2: Guide tree is built twice (2) in the progressive stage. - --inputorder: Order of sequences in the alignment is the same as their order in the input file. - [input file (Linux)] is the fasta file to align. The file name uses the Linux '/' rather than the Windows '\' separators. - [results file (Linux)] alignment export file name. The file name uses the Linux '/' rather than the Windows '\' separators. |
| Muscle | muscle5.1.win64.exe -align <input file> -output <results file>  where:   - <input file> is the fasta file to align - <results file > is the name of the file to save the alignment too. |
| PRANK | prank.exe -d=[input file (Linux)] -o=[results file (Linux)]  where:   - [input file (Linux)] is the fasta file to align. The file name uses the Linux '/' rather than the Windows '\' separators. - [results file (Linux)] alignment export file name. The file name uses the Linux '/' rather than the Windows '\' separators. |
| GBlocks | GBlocks.exe [input file] -t=[sequence type] -e=.fa  where:   - [input file] is the name, with the path of the original alignment file. - -t=[sequence type] indicates the sequence type (-t=d for DNA and -t=p for protein). - -e=.fa directs GBlocks to name the cleaned alignment to a file with the same name as the input file to which '.fa' has been appended. |

Table 2: The default commands used to automate each aligner and GBlocks
